# Prior information-assisted integrative analysis of multiple datasets

**DOI:** 10.1093/bioinformatics/btad452

**Published:** 2023-07-25

**Authors:** Feifei Wang, Dongzuo Liang, Yang Li, Shuangge Ma

**Affiliations:** Center for Applied Statistics, Renmin University of China, Beijing 100872, China; School of Statistics, Renmin University of China, Beijing 100872, China; Institute for Data Science in Health, Renmin University of China, Beijing 100872, China; School of Statistics, Renmin University of China, Beijing 100872, China; RSS and China-Re Life Joint Lab on Public Health and Risk Management, Renmin University of China, Beijing 100872, China; Center for Applied Statistics, Renmin University of China, Beijing 100872, China; School of Statistics, Renmin University of China, Beijing 100872, China; RSS and China-Re Life Joint Lab on Public Health and Risk Management, Renmin University of China, Beijing 100872, China; Department of Biostatistics, Yale University, New Haven, CT 06520, United States

## Abstract

**Motivation:**

Analyzing genetic data to identify markers and construct predictive models is of great interest in biomedical research. However, limited by cost and sample availability, genetic studies often suffer from the “small sample size, high dimensionality” problem. To tackle this problem, an integrative analysis that collectively analyzes multiple datasets with compatible designs is often conducted. For regularizing estimation and selecting relevant variables, penalization and other regularization techniques are routinely adopted. “Blindly” searching over a vast number of variables may not be efficient.

**Results:**

We propose incorporating prior information to assist integrative analysis of multiple genetic datasets. To obtain accurate prior information, we adopt a convolutional neural network with an active learning strategy to label textual information from previous studies. Then the extracted prior information is incorporated using a group LASSO-based technique. We conducted a series of simulation studies that demonstrated the satisfactory performance of the proposed method. Finally, data on skin cutaneous melanoma are analyzed to establish practical utility.

**Availability and implementation:**

Code is available at https://github.com/ldz7/PAIA. The data that support the findings in this article are openly available in TCGA (The Cancer Genome Atlas) at https://portal.gdc.cancer.gov/.

## 1 Introduction

In the past decades, genetic studies have been extensively conducted on multiple diseases and have led to significant advancements in understanding disease biology and clinical treatment. However, limited by sequencing cost and sample availability, genetic studies often suffer from the “small sample size, high dimensionality” problem (Liu *et al.* 2017). Additionally, it has been recognized that most of the genetic variables measured are “noises.” As such, it is critical to conduct variable selection along with regularized estimation. Many variable selection and dimension reduction methods have been applied to genetic studies (Wang *et al.* 2007, Li and Li 2008, Liu and Wong 2019). For many “common” scientific problems, there are often existing studies with similar goals and study designs. Accordingly, previous studies can potentially provide valuable “prior information” to the present study. A “straightforward” approach may be Bayesian (Van De Wiel *et al.* 2016, Zhao *et al.* 2019). Recent studies show that penalization and other regularization techniques can also provide effective solutions (Jiang *et al.* 2016, Wang *et al.* 2019). For example, Jiang *et al.* (2016) developed the prior LASSO method to select important single-nucleotide polymorphisms (SNPs) for bipolar disorder. To extract prior information, they first identified genes reported in previous biological/biomedical studies and then used SNPs in those genes to form the set of previously identified variants. Wang *et al.* (2019) developed a sparse group MCP method for gene–environment interaction analysis. To find prior information on the associations between genes and a disease, they searched PubMed and counted the number of articles containing both the genes and disease.

The information extraction methods used in the aforementioned and other published studies are easy to understand, simple to apply, and have led to sensible performance. However, as recognized in those studies, they have limitations. For example, Yuan *et al.* (2020) conducted a study on the mechanism of gender disparity in cutaneous melanoma incidence. Thirteen SNPs in four genes (ESR1, ESR2, IGF1, and IGF1R) were selected for candidate gene association analysis. However, only two SNPs in genes IGF1 and IGF1R were verified as relevant for melanoma risk. The other two genes, ESR1 and ESR2, were not considered to be relevant—however, as they did appear in the article, they might be mistakenly included in prior information by the existing methods. Thus, how to extract information more accurately from previous studies poses an important and challenging question for information-incorporated analysis. In recent years, multiple sophisticated text mining and machine learning techniques have emerged (Quan *et al.* 2014, Zeng *et al.* 2014), which can potentially provide new solutions.

In this and other published studies, prior information corresponds to existing studies that only provide summary findings but not raw data. Quite often, some raw data are also available, making it possible to collectively analyze multiple datasets to improve power/performance. Among the available multi-datasets analysis techniques, integrative analysis has emerged as an appealing choice (Ma *et al.* 2011, Liu *et al.* 2014, Huang *et al.* 2017, Li *et al.* 2022a). For example, it can be more flexible than pooled analysis (i.e. stacking all datasets directly) and more powerful than meta-analysis. Examples of integrative analysis include Liu *et al.* (2014), which proposed an integrative method with composite penalization for marker selection with four cancer datasets. Li *et al.* (2022a) proposed an integrative method with a functional SCAD penalty to analyze genome-wide association studies with multiple traits. It is noted that, in previously published integrative analysis, “prior information has not been incorporated.”

In this article, we develop a prior information-assisted method for the integrative analysis of multiple genetic datasets. It is noted that the proposed method can also be applied to other omics data and other scientific contexts. The proposed analysis consists of two steps. In the first step, the goal is to accurately extract prior information from existing literature. For a given research problem of interest (e.g. skin cutaneous melanoma—SKCM—in our numerical study), the goal is to identify important variables (in this case, genes) that have been reported as relevant to the disease. To this end, we first collect sentences containing genes and the disease in previously published articles. Then an active learning process (Settles 2009) with a deep neural network classifier (Kim 2014) is applied to assist labeling whether a gene is related to the disease in each sentence. With all sentences labeled, the associations between genes and the disease can be summarized and served as the prior information. In the second step, we integrate multiple datasets together and conduct regularized estimation and gene selection using group LASSO. To incorporate the extracted prior information, we follow the strategy of Jiang *et al.* (2016) to adjust the penalty according to the prior information. Finally, we demonstrate the model performance by extensive simulations as well as a real SKCM dataset.

Although sharing some building blocks with the existing literature, this study can advance in the following important ways. First, to the best of our knowledge, it is the first to incorporate prior information in integrative analysis, further expanding its scope. Although it may seem “natural” to extend the strategy of Jiang *et al.* (2016) and others, this has not been pursued in the existing literature. Second, this study delivers a new prior information extraction method, which can be much more refined than those in relevant literature. It is noted that the proposed prior information extraction can also be coupled with other analysis schemes/methods. Third, with the popularity of integrative analysis and with the fast accumulation of prior information, this study can provide a practically highly useful tool—this can be especially true with the development of software codes ready to be used.

## 2 Materials and methods

### 2.1 Analysis framework

**Figure 1.**
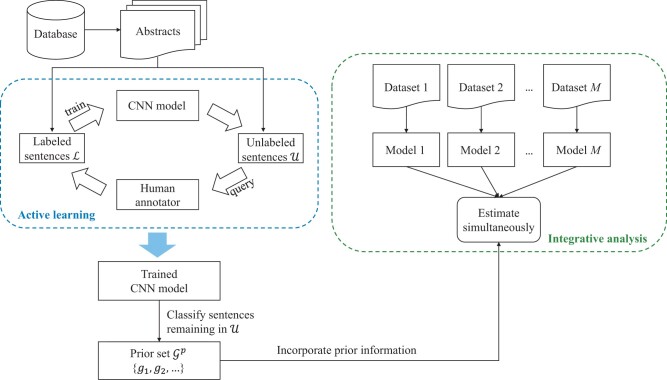
Overall analysis framework.

In the integrative analysis of multiple genetic datasets, usually variable selection is of essential interest (with the consideration that, once relevant genetic variables are properly selected, model building can be straightforward). Assume that there are *M* datasets from independent studies. Each dataset contains a response variable (e.g. Breslow thickness for SKCM) and *p* covariates (e.g. gene expressions). Denote the *p* covariates as $G=\{g_{1},g_{2},\ldots ,g_{p}\}$. To take advantage of information contained in published literature, we develop a novel approach to extract gene–disease association information and then incorporate it in downstream analysis. Then, to effectively increase sample size and power, we integratively analyze multiple datasets and apply penalization for variable selection and estimation. The overall analysis framework is graphically presented in Fig. 1.

The proposed method consists of two main steps. In the first step, the goal is to obtain high-quality information from previous literature. To this end, we first prepare the dataset that will be used to extract prior information. Specifically, we collect abstracts of published articles in PubMed, in which the disease and genes in G have co-occurred. The proposed analysis can be easily extended to mining full articles. We further narrow the searching scope down to the sentence level and filter out all sentences containing the disease and genes. Different from some published studies, here we note that sometimes genes and a disease mentioned in the same sentences are not related. When there are a few abstracts, finer results can be generated manually. However, this is not possible when there are a large number of abstracts. To this end, we treat this as a classification problem and apply a convolutional neural network (CNN) model to automatedly distinguish gene–disease relationships. To reduce the cost of manual labeling, we propose an active learning approach to train the CNN, which applies a progressively labeling strategy to enhance classification performance. By summarizing the results from the CNN, we can find the correlation strength between the disease and each gene.

In the second step, to describe the relationship between the disease and genes, we first assume a statistical model for each dataset. In our data analysis, the response variable is continuous, and hence we assume linear regression models, which can be replaced by other regression models. In principle, the proposed analysis can accommodate mismatched gene sets (Shi *et al.* 2014). To simplify notation, we assume the same set of genes for all datasets. For variable selection and regularized estimation, we adopt group LASSO. To incorporate the extracted prior information, we follow the strategy of Jiang *et al.* (2016). Below, we provide detailed descriptions in Sections 2.2 and 2.3.

### 2.2 Prior information extraction

Information generated by previous studies is stored in many different sources. Here, we focus on publications, which may contain higher-quality information. We further focus on abstracts of publications. Beyond the consideration that some literature databases not always have full-length articles, we also recognize that abstracts may contain the most relevant information in a highly concise manner. Focusing on abstracts is a notable difference/advancement of this study. We fully acknowledge the “publication bias” problem. However, it is still well recognized that information in publications can be overall useful. In the proposed analysis, prior information is used to assist, not dominate, analysis in the sense that the proposed approach can flexibly accommodate incorrect (both false positive and false negative) prior information. More detailed discussions are provided in Supplementary Material S7.

We first download all abstracts that include the disease of interest from a literature database. Denote the abstract set as $D=\{d_{1},d_{2},\ldots \}$. Then, for each abstract in D, we split into sentences and only keep those that contain the disease and at least one gene in G. The remaining sentence set is denoted by $S=\{s_{1},s_{2},\ldots \}$.

Quantifying disease–gene relationships can be viewed as a binary classification problem (i.e. whether a gene and the disease are related or not). When there are a small number of abstracts, this can be done manually. However, this can be too costly when there are a large number of abstracts. Here, we propose first labeling only a small proportion of the sentences and then applying machine learning to classify the rest of the sentences. Among the available machine learning techniques, deep learning has emerged as highly competitive. In this study, we adopt the CNN technique (Kim 2014) to classify the sentences. The architecture of the CNN model is presented in Supplementary Material S1.

Usually, to train a CNN model with high accuracy, a large training data are needed. To reduce the number of labeled sentences and save effort, we propose adopting an active learning strategy. Active learning is an iterative cyclic process between an oracle (e.g. an expert annotator) and an active learner (e.g. a machine learning model). The goal of active learning is to achieve high accuracy using as few labeled data as possible, thereby minimizing the cost of labeling data (Settles 2009). As a start, we select a few (say 200) sentences from S and manually label whether a gene is relevant to the disease in each sentence. Then the sentence set S can be divided into “labeled” sentences L and “unlabeled” sentences U. The labeled sentence set L is evenly split into two parts: the initial training set $L_{\text{training}}$ and the test set $L_{\text{test}}$, which will be used to evaluate model performance. After transferring all sentences into word vectors using embedding techniques, we start the active learning process by iteratively training the CNN model and labeling more data. Specifically, we first train the CNN model using the sentences currently in $L_{\text{training}}$. Then we use the trained model to predict labels of the sentences in U. For each unlabeled sentence in U, the CNN model can predict its relevancy probability. The sentences whose relevancy probabilities are around 0.5 are hard to be classified. To address this problem, we manually label these sentences, add them in $L_{\text{training}}$, and remove them from U. The resulting $L_{\text{training}}$ can then be used to train a new CNN model. We repeat this process until model performance on the test set does not further improve. Then the final CNN model is regarded as the optimal one. The whole active learning process is summarized in Algorithm 1.

**Algorithm 1.** Active learning with CNN

**Require: ** labeled sentence set L; unlabeled sentence set U

**Ensure: ** optimal CNN model

1: ** **Convert sentences in L and U into word vectors, and initiate the CNN model;

2: ** **Split L into $L_{\text{training}}$ and $L_{\text{test}}$ according to a prespecified ratio;

3: ** repeat**

4: **  **Train the CNN model with sentences in $L_{\text{training}}$;

5: **  **Calculate prediction accuracy on test set $L_{\text{test}}$;

6: **  **Predict relevancy probabilities for sentences in U;

7: **  **Label sentences whose relevancy probabilities are around 0.5 (denoted as $U^{*}$);

8: **  **Update $U\leftarrow U\setminus U^{*}$ and $L_{\text{training}}\leftarrow L_{\text{training}}\cup U^{*}$;

9: ** until** model performance on $L_{\text{test}}$ no longer improves

10: ** return** the optimal CNN model

We then applied the resulted optimal CNN model to predict labels for sentences in U. For the *j*th gene (1≤j≤p), we calculate the number of sentences that are classified as related to the disease and denote this count as *c_j_*. A larger value of *c_j_* indicates more evidence for the relevancy between the disease and gene *g_j_* in the literature. We construct the prior set as $G^{p}=\{g_{j}|c_{j}\geq a,j=1,2,\ldots ,p\}$, where *a* is a prespecified threshold. The prior set $G^{p}$ will then be used in integrative analysis in the next section. We summarize the process of obtaining $G^{p}$ in a flowchart in Supplementary Material S2.

### 2.3 Prior information-assisted integrative analysis

With *M* datasets, for the *m*-th dataset, let $Y^{\left(m\right)}={\left(Y_{1}^{\left(m\right)},\ldots ,Y_{n^{\left(m\right)}}^{\left(m\right)}\right)}^{\prime }\in R^{n^{\left(m\right)}}$ be the continuous response vector, where $n^{\left(m\right)}$ is the sample size. Let $X^{\left(m\right)}=(X_{1}^{\left(m\right)},\ldots ,X_{p}^{\left(m\right)})\in R^{n^{\left(m\right)}\times p}$ be the design matrix for the *p* covariates in G, where $X_{j}^{\left(m\right)}={\left(X_{j1}^{\left(m\right)},\ldots ,X_{jn^{\left(m\right)}}^{\left(m\right)}\right)}^{\prime }$ is the realization of covariate *g_j_*. Assume that both the response and covariates are centralized. Then, to model the relationship between the response and *p* covariates, we consider the model: where $\beta^{\left(m\right)}={\left(\beta_{1}^{\left(m\right)},\ldots ,\beta_{p}^{\left(m\right)}\right)}^{\prime }\in R^{p}$ is the regression coefficient vector, $\varepsilon^{\left(m\right)}={\left(\varepsilon_{1}^{\left(m\right)},\ldots ,\varepsilon_{n^{\left(m\right)}}^{\left(m\right)}\right)}^{\prime }\in R^{n^{\left(m\right)}}$ is the error vector with marginal means zero and covariance matrix $\sigma^{2}I_{n^{\left(m\right)}}$, and $I_{n^{\left(m\right)}}$ stands for the $n^{\left(m\right)}$-dimensional identity matrix.


(1) $$Y^{\left(m\right)}=X^{\left(m\right)}\beta^{\left(m\right)}+\varepsilon^{\left(m\right)},$$

Let $n=\sum_{m=1}^{M}n^{\left(m\right)},\;Y={\left(Y^{\left(1\right)}{}^{\prime },\ldots ,Y^{\left(M\right)}{}^{\prime }\right)}^{\prime }\in R^{n},\;X=\mathrm{diag}(X^{\left(1\right)},\ldots ,X^{\left(M\right)})\in R^{n\times Mp},\;\beta ={\left(\beta^{(1)\prime },\ldots ,\beta^{(M)\prime }\right)}^{\prime }\in R^{Mp}$, and $\varepsilon ={\left(\varepsilon^{\left(1\right)}{}^{\prime },\ldots ,\varepsilon^{\left(M\right)}{}^{\prime }\right)}^{\prime }\in R^{n}$. Then we can rewrite (1) as:


(2) Y=Xβ+ε˙

**Figure 2.**
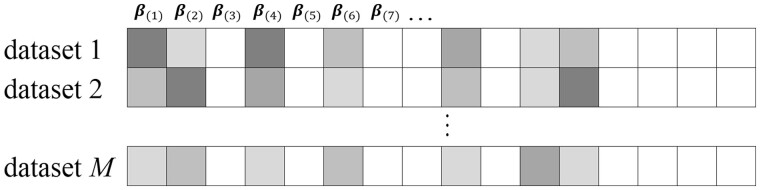
Sparsity structures for the *M* datasets. The white rectangles represent zero coefficients, and the shaded rectangles with different gray levels represent different non-zero coefficients.

In variable selection, we assume the homogeneity model (Liu *et al.* 2014, Huang *et al.* 2017), under which the *M* datasets share the same set of important variables. In the literature, an alternative is the heterogeneity model. We expect that the proposed analysis can be extended to the heterogeneity model by choosing a two-level selection penalty—this is deferred to future research. Here, it is stressed that although the *M* models have the same sparsity structure, their individual coefficients can be different (Fig. 2).

We then follow the strategy of Jiang *et al.* (2016) to incorporate the prior information into integrative analysis. First, assume that the prior information is fully credible. Then in model (2), covariates in $G^{p}$ are automatically included, and variable selection is conducted with the rest of the variables. Specifically, the loss function is defined as: where $\beta_{\left(j\right)}=(\beta_{j}^{\left(1\right)},\ldots ,\beta_{j}^{\left(M\right)})\prime$, *κ* is a tuning parameter, and $||\cdot |{|}_{2}$ is the *L*_2_ norm. Here, if needed, *a* can be adjusted in a way that $G^{p}$ is smaller than the sample size. Denote $\hat{\dot{\beta }}=\arg\min_{\beta }L_{\kappa ,G^{p}}\left(\beta ;X,Y\right)$, and we can calculate the predicted values as $\hat{\dot{Y}}=X\hat{\dot{\beta }}$.


(3) $$\begin{array}{cc} & L_{\kappa ,G^{p}}\left(\beta ;X,Y\right)\\ = & {\left(Y-X\beta \right)}^{\prime }\left(Y-X\beta \right)+\kappa \sum_{j=1}^{p}{\left\|\beta_{\left(j\right)}\right\|}_{2}I(g_{j}\notin G^{p}), \end{array}$$

It is not uncommon that findings in previous studies are only partially correct or even wrong. As such, $G^{p}$ may not be fully credible. Additionally, the present data may differ from those in published studies. It is thus desirable to “balance” between the present data and prior information. To this end, we consider the following loss function: where $\tilde{Y}=(Y+\eta \hat{\dot{Y}})/(1+\eta )$. The detailed deviation is provided in Supplementary Material S3. The loss function contains three parts. The first part L(β;X,Y) represents the original information from data. The second part $L(\beta ;X,\hat{\dot{Y}})$ represents information from the prior set. The third part is the penalty term. *λ* and *η* are tuning parameters, with *η* balancing the relative importance of data and prior information and *λ* representing the penalty level. A larger value of *η* corresponds to a higher quality of the prior information. In numerical studies, all tuning parameters are selected by cross-validation. By derivations in (4), we construct a new response $\tilde{Y}$, which combines the data information and prior information. Then the resulted loss function has the same form as penalized integrative analysis methods (Zhao *et al.* 2015), and we can apply similar computational algorithms. The final estimator is defined as $\hat{\beta }=\arg\min_{\beta }L_{\lambda ,\eta }(\beta ;X,Y,\hat{\dot{Y}})$. A group coordinate descent (Breheny and Huang 2015) algorithm is described in Supplementary Material S4.


(4) $$\begin{array}{cc} & L_{\lambda ,\eta }(\beta ;X,Y,\hat{\dot{Y}})\\ = & L(\beta ;X,Y)+\eta L(\beta ;X,\hat{\dot{Y}})+\lambda \sum_{j=1}^{p}{\left\|\beta_{\left(j\right)}\right\|}_{2}\\ = & {\left(Y-X\beta \right)}^{\prime }(Y-X\beta )+\eta {\left(\hat{\dot{Y}}-X\beta \right)}^{\prime }(\hat{\dot{Y}}-X\beta )+\lambda \sum_{j=1}^{p}{\left\|\beta_{\left(j\right)}\right\|}_{2}\\ \propto & {\left(\tilde{Y}-X\beta \right)}^{\prime }\left(\tilde{Y}-X\beta \right)+\frac{\lambda }{1+\eta }\sum_{j=1}^{p}{\left\|\beta_{\left(j\right)}\right\|}_{2}, \end{array}$$

To facilitate routine utilization, we develop software codes and make them publicly available at GitHub. Detailed information is provided in Supplementary Material S5.

## 3 Simulation

### 3.1 Active learning

To evaluate the performance of the active learning strategy, we consider the logistic regression model as an example. In this example, we consider *p* = 8 covariates $X_{i}={\left(X_{i1},\ldots ,X_{i8}\right)}^{\prime }$ for the *i*-th sample. The first four covariates are discrete, each of which is generated from a Bernoulli distribution with probability 0.5. The last four covariates are continuous and generated from a multivariate normal distribution with marginal means zero and covariance matrix $\Sigma =(\sigma_{ij})$, where $\sigma_{ii}=1$ and $\sigma_{ij}=0.5$. The corresponding coefficient β is ${\left(-0.75,0.5,-0.75,-0.5,-1,-0.75,0.5,-1\right)}^{\prime }$. *Y_i_* is generated from a Bernoulli distribution with probability $1/(1+e^{-z_{i}})$, where $z_{i}=\beta_{0}+\sum_{j=1}^{8}\beta_{j}X_{ij}$ and $\beta_{0}=0.75$.

The generated dataset is randomly split into a labeled set L and an unlabeled set U. Specifically, we generate a total of *N* = 10 000 samples, and the labeled set L contains 100 samples and the unlabeled set U contains 9900 samples. The labeled set L is used to initialize the classifier. Then we start the active learning process by iteratively selecting samples from the unlabeled set U and retrain the classifier. Prediction performance is evaluated using the whole unlabeled set U. For comparison, the random sampling strategy is also conducted, in which the samples are randomly selected from U to enhance the classifier.

**Figure 3.**
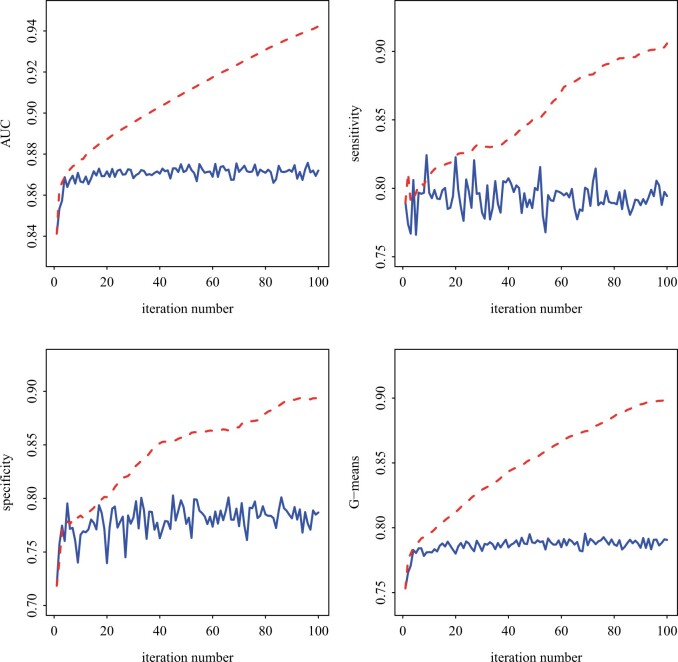
Simulation results for the active learning (dashed line) and random sampling strategies (solid line).

Four measures are used for evaluation: (i) AUC, (ii) sensitivity, (iii) specificity, and (iv) G-means, which is defined as $\sqrt{\text{sensitivity}\times \text{specificity}}$. For all four measures, bigger values indicate better classification performance. We conduct a total of 100 iterations with each iteration selecting 50 samples in active learning. The simulation results are present in Fig. 3. We observe improved performance over iterations for active learning, but not random sampling. These results suggest that the active learning strategy can quickly achieve a satisfactory classifier. For example, after 50 iterations, the classifier under the active learning strategy has an AUC of 0.90 and G-means of 0.85. Consequently, the labels in U can be more accurately predicted.

### 3.2 Integrative analysis

We consider *M* = 3 independent datasets, each with a sample size of 100. All the datasets share the same *p* = 1000 covariates. For each sample, its covariate vector $X={\left(X_{1},\ldots ,X_{p}\right)}^{\prime }$ is generated from a multivariate normal distribution with marginal means zero and covariance matrix $\Sigma =(\sigma_{ij})$, where $\sigma_{ij}=\rho^{\left|i-j\right|}$ for 1≤i,j≤p and ρ=0.5. For the three regression coefficient vectors, we set:


$$\begin{array}{c} \beta^{\left(1\right)}=(-0.5,-2,0.5,2,-1.5,1,2,-1.5,2,-2,1,\\ 1.5,-2,1,1.5,0,\ldots ,0)\prime ,\\ \beta^{\left(2\right)}=(1,1.5,-2,1,1.5,-0.5,-2,\\ 0.5,2,-1.5,1,2,-1.5,2,-2,0,\ldots ,0)\prime ,\\ \beta^{\left(3\right)}=(1,2,-1.5,2,-2,1,1.5,-2,1,1.5,-0.5,-2,0.5,\\ 2,-1.5,0,\ldots ,0)\prime ˙ \end{array}$$

Further we generate random errors independently from a normal distribution $N(0,3^{2})$. Then the response *Y* is calculated as $Y=X^{\prime }\beta^{\left(m\right)}+\varepsilon$. We repeat the data generation process for *B* = 100 times. To examine prediction performance, we use the same process to generate three independent test sets, each of which has a sample size of 10 000.

To investigate the impact of prior information quality on variable selection and prediction, we consider six settings. They are $G_{1}^{p}=\{g_{1},\ldots ,g_{5}\},\;G_{2}^{p}=\{g_{1},\ldots ,g_{10}\},\;G_{3}^{p}=\{g_{1},\ldots ,g_{15}\},\;G_{4}^{p}=\{g_{1},\ldots ,g_{10}\}\cup \{g_{16},\ldots ,g_{20}\},\;G_{5}^{p}=\{g_{1},\ldots ,g_{10}\}\cup \{g_{16},\ldots ,g_{25}\},\;G_{6}^{p}=\{g_{1},\ldots ,g_{10}\}\cup \{g_{16},\ldots ,g_{60}\}$. $G_{1}^{p},\;G_{2}^{p}$, and $G_{3}^{p}$ only include prior information for the truly relevant covariates. $G_{4}^{p},\;G_{5}^{p}$, and $G_{6}^{p}$ contain prior information for both relevant and irrelevant covariates. More settings can be found in Supplementary Material S6.2.

For each generated dataset, we apply the proposed method (denoted by M1) and three alternatives denoted as M2, M3, and M4. M2 conducts integrative analysis without incorporating prior information. M3 stacks the *M* datasets and applies the prior LASSO method. M4 applies prior LASSO for estimation with each dataset separately. Comparing M1 with M2 can establish the merit of incorporating prior information, and comparing with M3 and M4 can establish the merit of integrative analysis.

We first examine variable selection performance measured by sensitivity (also called recall), specificity, precision, and G-means. The results are summarized in Table 1. Under all the simulation settings, M1 achieves the highest values in sensitivity and comparable values in specificity. When measured by precision, M1 outperforms M2 and M4 but has a worse performance than M3. These results suggest that M1 selects more relevant covariates but also involves more noises than M3. When further measured by the overall criterion G-means, M1 is always the best. M3 generates the same estimation results for all datasets and thus cannot reflect the differences across datasets. Finally, we compare selection performance under different prior information settings. When comparing $G_{1}^{p}$ to $G_{3}^{p}$, we see an increasing trend for all evaluation metrics. This finding suggests that more truly relevant variables contained in the prior set can result in better variable selection. On the other hand, with more irrelevant variables contained in the prior set (as with $G_{4}^{p}$ to $G_{6}^{p}$), variable selection can be worse.

**Table 1.** Simulation results for variable selection.

Prior set	M1		M2		M3		M4	
	Mean	SD	Mean	SD	Mean	SD	Mean	SD
Sensitivity								
$G_{1}^{p}$	0.647	0.087	0.488	0.119	0.246	0.098	0.309	0.066
$G_{2}^{p}$	0.739	0.091	0.488	0.119	0.317	0.110	0.341	0.076
$G_{3}^{p}$	0.827	0.097	0.488	0.119	0.416	0.130	0.354	0.089
$G_{4}^{p}$	0.721	0.096	0.488	0.119	0.323	0.112	0.320	0.074
$G_{5}^{p}$	0.698	0.107	0.488	0.119	0.323	0.112	0.313	0.071
$G_{6}^{p}$	0.561	0.129	0.488	0.119	0.298	0.110	0.245	0.072
Specificity								
$G_{1}^{p}$	0.994	0.004	0.994	0.005	0.997	0.004	0.993	0.003
$G_{2}^{p}$	0.994	0.002	0.994	0.005	0.998	0.003	0.992	0.004
$G_{3}^{p}$	0.994	0.002	0.994	0.005	0.997	0.003	0.992	0.004
$G_{4}^{p}$	0.993	0.003	0.994	0.005	0.997	0.003	0.992	0.004
$G_{5}^{p}$	0.993	0.003	0.994	0.005	0.997	0.003	0.992	0.004
$G_{6}^{p}$	0.993	0.005	0.994	0.005	0.994	0.005	0.993	0.004
Precision								
$G_{1}^{p}$	0.641	0.123	0.621	0.158	0.712	0.238	0.440	0.133
$G_{2}^{p}$	0.672	0.090	0.621	0.158	0.759	0.203	0.432	0.108
$G_{3}^{p}$	0.704	0.085	0.621	0.158	0.768	0.183	0.422	0.100
$G_{4}^{p}$	0.641	0.093	0.621	0.158	0.725	0.218	0.417	0.099
$G_{5}^{p}$	0.624	0.095	0.621	0.158	0.687	0.201	0.411	0.103
$G_{6}^{p}$	0.620	0.154	0.621	0.158	0.522	0.210	0.369	0.124
G-means								
$G_{1}^{p}$	0.800	0.053	0.691	0.084	0.483	0.111	0.550	0.060
$G_{2}^{p}$	0.856	0.053	0.691	0.084	0.553	0.100	0.578	0.065
$G_{3}^{p}$	0.905	0.055	0.691	0.084	0.636	0.104	0.587	0.077
$G_{4}^{p}$	0.845	0.057	0.691	0.084	0.558	0.102	0.559	0.067
$G_{5}^{p}$	0.830	0.065	0.691	0.084	0.558	0.101	0.554	0.063
$G_{6}^{p}$	0.741	0.086	0.691	0.084	0.535	0.101	0.487	0.072

**Figure 4.**
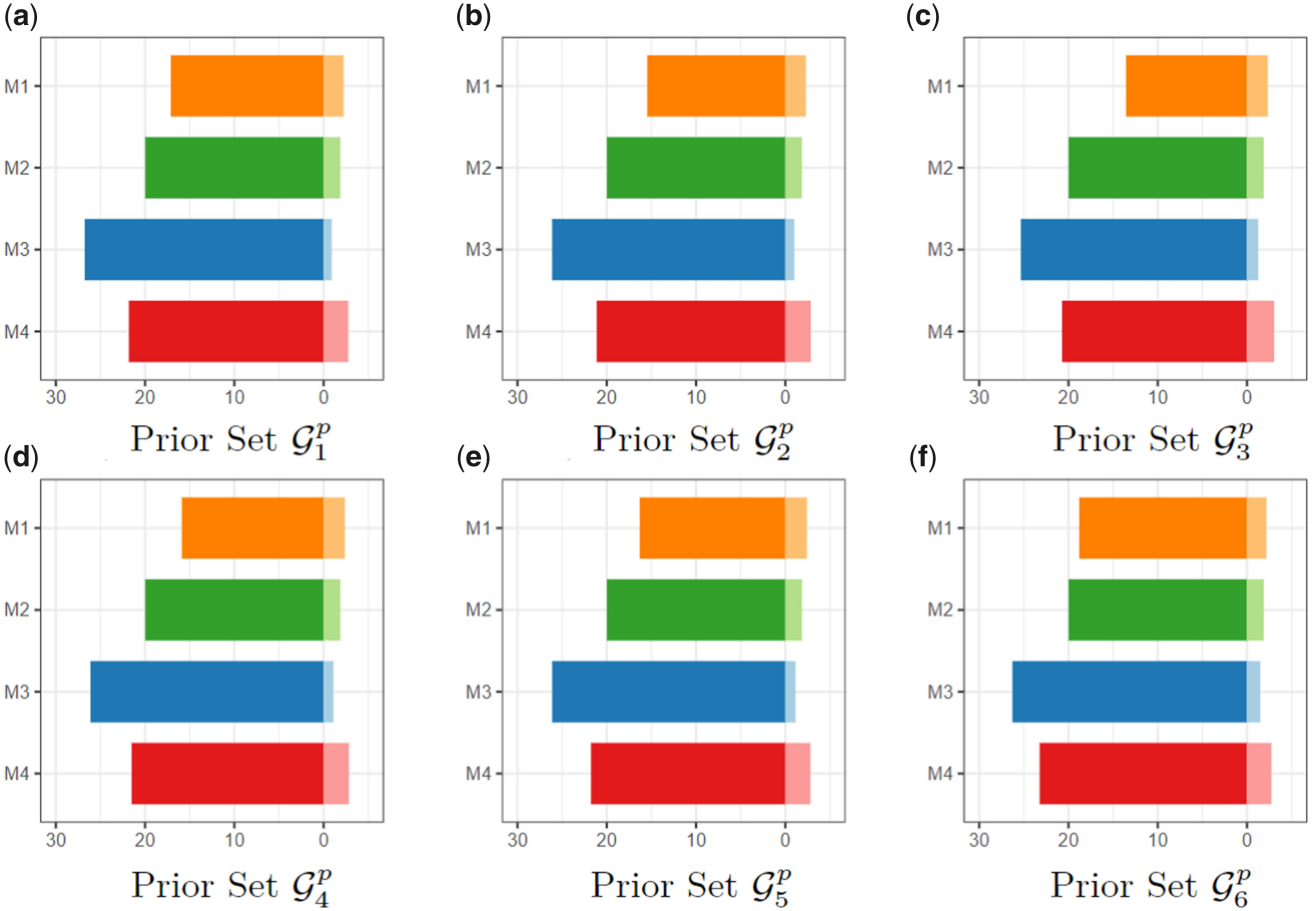
Simulation results for prediction. The overall bar represents MSE, with the square of bias on the left and variance on the right.

When evaluating prediction performance, we first compute the mean squared error (MSE) on the test sets. We also split MSE into the square of bias and variance. The former evaluates prediction accuracy, and the latter evaluates prediction stability. The results are present in Fig. 4. We observe that M1 always has the smallest MSE. The four methods have similar variances, while M1 has the lowest square of bias.

Next, we evaluate performance of parameter estimation. We take the prior information setting $G_{1}^{p}$ as an example. Supplementary Material S6.1 contains the heatmaps of the estimated coefficients for $\beta^{\left(1\right)},\;\beta^{\left(2\right)}$, and $\beta^{\left(3\right)}$ in each generated dataset. Each heatmap has 50 rows (the first 50 variables) and *B* = 100 columns, and the (*i*, *j*)-th position represents the estimate of $\beta_{51-i}^{\left(m\right)}$ in the *j*-th simulated data. As the first 15 coefficients are truly non-zero, we expect the bottom of the heatmaps to have different colors (representing the non-zero coefficients), while the upper part to be white (representing the zero coefficients). As shown in Supplementary Material S6.1, the heatmaps of M1 and M2 are similar. Their bottom parts show consistent patterns across replications, and the upper parts are almost white. These results indicate that M1 and M2 can select the truly relevant covariates and exclude the irrelevant ones. For M3 and M4, although the upper parts of their heatmaps are also almost white, the bottom parts have many white points. These results imply that M3 and M4 cannot identify all the relevant covariates stably. It is also noted that the three heatmaps for M3 are the same, as M3 pools all datasets together and analyze them as a whole.

## 4 Analysis of SKCM data

SKCM is one of the most aggressive malignancies, and its incidence has been increasing. To identify important genes relevant to SKCM, we collect data from The Cancer Genome Atlas (TCGA) (https://www.cancer.gov/about-nci/organization/ccg/research/structural-genomics/tcga). This dataset consists of patients in six clinical stages. The total sample size is 347, with 78 in Stage I, 129 in Stage II, 123 in Stage III, 10 in Stage IV, 4 in Stage V, and 3 in Stage VI. We merge the last four stages together, which has sample size 140—with a slight abuse of terminology, we still refer to it as Stage III. Since all samples in the SKCM dataset are collected under the same protocol, similarity is expected, which forms the basis of integrative analysis. Furthermore, from some preliminary analysis, we find that genes in different stages have different effects. Thus, the three stages are treated as three separate “studies” and will be analyzed in integrative analysis. This strategy has been adopted in the published studies (Liang *et al.* 2021, Li *et al.* 2022b). In the SKCM dataset, the response variable is “Breslow thickness,” a measurement of the depth of melanoma and strongly related to prognosis. A total of 18 335 gene expressions are measured for each sample.

**Figure 5.**
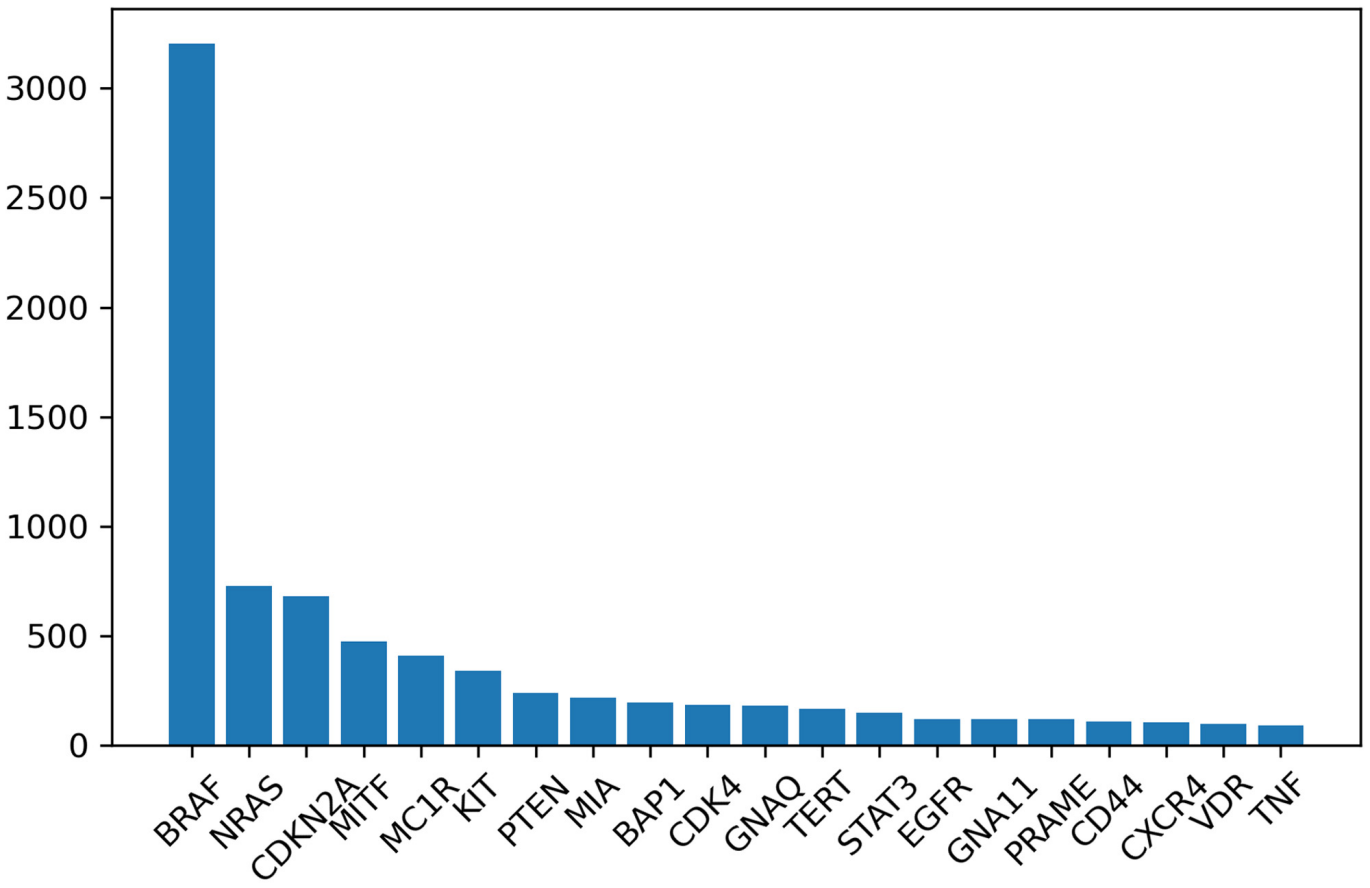
The top 20 genes with the highest counts.

Prior information extraction is conducted using the proposed approach. More details are provided in Supplementary Material S2 and S8.1. Briefly, the abstracts of 111 102 articles related to melanoma are obtained from PubMed. We identify 26 266 sentences containing genes and cutaneous melanoma. With this active learning procedure, 13 454 sentences are labeled as relevant, and 2018 genes are contained in those sentences. We further count the number of relevant sentences for each gene. By drawing a scatter plot of gene counts in a descending order, we find an elbow point at the top 20 genes. The detailed counts of those genes are shown in Fig. 5. Those genes are used to construct the prior set $G^{p}$.

To improve the stability of estimation, we conduct marginal screening and select the top 1000 genes with the highest absolute correlation coefficients for downstream analysis. This preprocessing step is commonly used in the literature. The 20-gene set is combined with this set. We then normalize each gene expression within each dataset separately. The response variable is log-transformed. The data are analyzed using the proposed and alternative methods. The numbers of genes selected by M1–M4 are 88, 106, 112, and 161, respectively. Detailed results are provided in Supplementary Material S8.3.

To make a more focused comparison, Table 2 only includes the selection results for the genes in the prior set. It is observed that not all genes in the prior information set are selected by the four methods. We see that M1 selects more genes in the prior information set than M2. This result again demonstrates the influence of prior information on gene selection. Compared to M3 and M4, M1 selects a more moderate number of genes. This may be because M3 and M4 select more irrelevant genes.

**Table 2.** Selected genes that are included the prior set.^a^

Gene	M1	M2	M3	M4		
	D1/2/3	D1/2/3	D1/2/3	D1	D2	D3
CDK4	*✓*		*✓*		*✓*	
CDKN2A	*✓*	*✓*	*✓*			*✓*
CXCR4	*✓*	*✓*	*✓*		*✓*	
GNA11	*✓*		*✓*		*✓*	*✓*
GNAQ	*✓*				*✓*	
MC1R	*✓*		*✓*		*✓*	*✓*
MIA	*✓*			*✓*	*✓*	*✓*
MITF	*✓*	*✓*	*✓*		*✓*	*✓*
NRAS	*✓*		*✓*		*✓*	*✓*
PRAME	*✓*		*✓*			*✓*
BAP1			*✓*			*✓*
BRAF			*✓*		*✓*	*✓*
CD44			*✓*			
KIT			*✓*		*✓*	*✓*
PTEN			*✓*			*✓*
TERT			*✓*		*✓*	
TNF			*✓*			
VDR			*✓*		*✓*	*✓*
EGFR						
STAT3						

a D1–D3 correspond to the three stages, and the check mark indicates genes selected by the method.

Literature search suggests that the identified genes can be biologically highly meaningful. For example, mutations in gene NRAS are among the most common mutations found in malignant melanoma. A prospective cohort study of 249 patients showed that NRAS mutations were associated with thicker tumors and higher rates of mitosis when compared to BRAF V600E and wild-type melanoma (Devitt *et al.* 2011). Without incorporating the prior information, M2 fails to identify gene NRAS. The variants of gene MC1R were frequently found in patients having melanomas at a younger age (McMeniman *et al.* 2020). A meta-analysis showed that seven variants of gene MC1R were significantly associated with SKCM (Raimondi *et al.* 2008). However, M2 fails to identify gene MC1R, and M4 also fails to identify it for Stage I. Another example is gene CDKN2A (p16) which is identified by all of the four methods. It has been verified as a melanoma susceptibility gene (Gruis *et al.* 1995). Targeted germline sequencing showed that patients with ≥3 primary melanomas had a high rate of pathogenic variants in gene CDKN2A (Li *et al.* 2020). However, M4 fails to identify it for the first two datasets. Note that some genes not included in the prior set have also been selected by M1. Among them, some genes (e.g. CDKN1A and PRMT6) have been suggested to be related to melanoma (Soto *et al.* 2005, Limm *et al.* 2013).

With practical data, there is a lack of way for objectively comparing selection performance. We resort to prediction evaluation for “indirect” support. Specifically, we randomly split data into a training set (80%) and a test set (20%). We apply the four methods to the training data and then use the training data models to make prediction for the test data. The MSE is computed. This process is repeated 50 times. The resulted 50 MSE values are plotted in Supplementary Material S8.2. The average MSE values are 1.459, 1.483, 1.905, and 1.526 for M1–M4, respectively. The competitive prediction performance can provide support to the validity of our analysis.

## 5 Conclusion and discussion

In this study, we have developed a two-step prior information-assisted integrative analysis method. In the first step, a CNN model with active learning has been proposed to extract comprehensive and accurate prior information from published studies. In the second step, the prior information has been incorporated for integrative variable selection with group LASSO. The connections between this work and published ones such as Jiang *et al.* (2016) are well noted. However, with the new prior information extraction approach, and with the integrative analysis of multiple datasets, this study is warranted beyond the existing literature. Extensive simulations and data analysis have demonstrated the practical utility of the proposed approach. Overall, this study can further advance the field by delivering a practically useful tool for genetic data analysis and marker identification.

This study can be extended in multiple directions. First, we currently extract prior information at the sentence level. Sentences from the same abstract may contain duplicative meanings, and the disease name may not be included in certain sentences. It may be possible to improve prior information construction by directly extracting information at the abstract level or using more advanced techniques to analyze full articles. Second, we use an empirical approach to select the number of covariates in the prior information set. More effective approaches will be worth investigating in the future. Third, for the integrative model, we assume that different datasets share the same set of important variables. This homogeneity assumption can be further relaxed. In addition, to better incorporate prior information in (3), we may consider, as an alternative, the sentence count *c_j_* as weight for each covariate in prior information to replace the current threshold. Last, the performance of our proposed method can be further investigated with more datasets.

## Supplementary Material

btad452_Supplementary_Data
