# Supplementary material for: Prior information-assisted integrative analysis of multiple datasets
Source: Bioinformatics. 2023 Jul 25;39(8):btad452. doi: 10.1093/bioinformatics/btad452 (PMC10400378; doi:10.1093/bioinformatics/btad452)
Supplement: btad452_Supplementary_Data [file btad452_supplementary_data.pdf]

Supplementary Materials for "Prior Information Assisted Integrative Analysis of Multiple Datasets"

S1 CNN architecture

Overall, the CNN model consists of one input layer, one convolutional layer with multiple kernels of three different sizes, one max pooling layer, and one output layer. The input of the CNN model is word embedding matrices which represent the sentences to be classified.

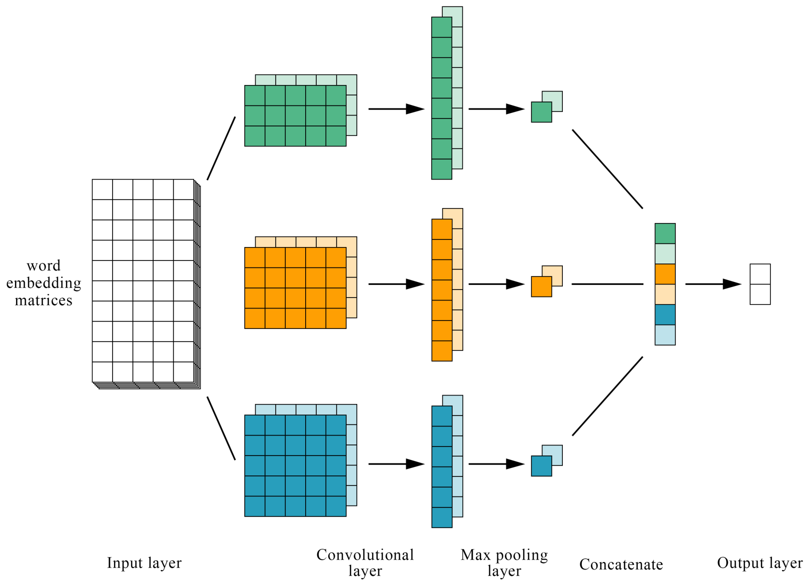

Figure S1: CNN architecture. For better illustration, only two kernels of each size are shown.

S2 Flowchart for prior set construction

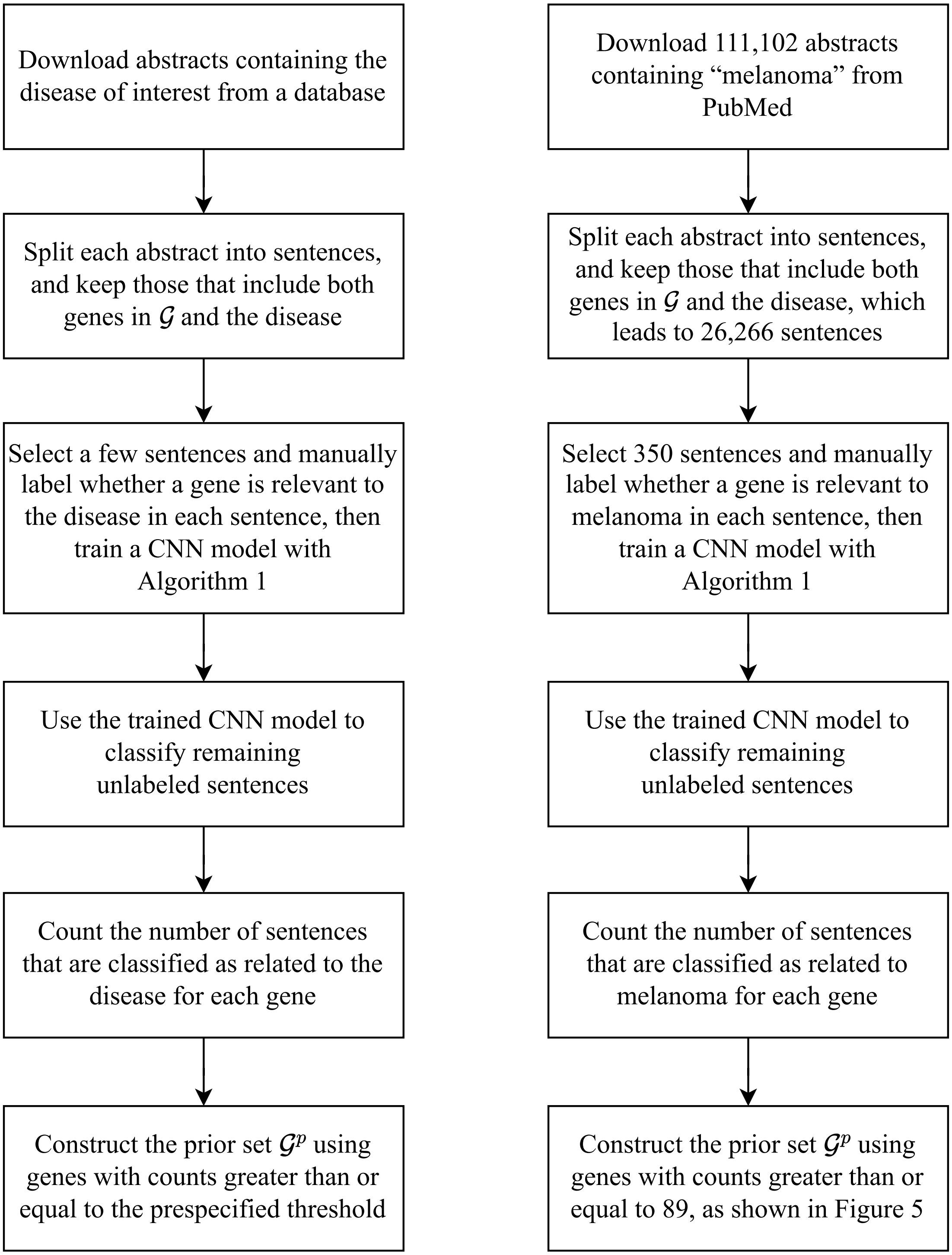

Figure S2: The general flowchart (left) and that for the SKCM data analysis (right).

S3 Detailed deviation of equation (4)

$$
\begin{aligned}
& L_{\lambda,\eta}(\boldsymbol{\beta};\mathbb{X},\mathbb{Y},\widehat{\dot{\mathbb{Y}}}) \\
=& L(\boldsymbol{\beta};\mathbb{X},\mathbb{Y}) + \eta L(\boldsymbol{\beta};\mathbb{X},\widehat{\dot{\mathbb{Y}}}) + \lambda \sum_{j=1}^{p} \left\|\boldsymbol{\beta}_{(j)}\right\|_2 \\
=& \big(\mathbb{Y}-\mathbb{X}\boldsymbol{\beta}\big)^{'}\big(\mathbb{Y}-\mathbb{X}\boldsymbol{\beta}\big) + \eta\big(\widehat{\dot{\mathbb{Y}}}-\mathbb{X}\boldsymbol{\beta}\big)^{'}\big(\widehat{\dot{\mathbb{Y}}}-\mathbb{X}\boldsymbol{\beta}\big) + \lambda \sum_{j=1}^{p} \left\|\boldsymbol{\beta}_{(j)}\right\|_2 \\
=& \mathbb{Y}^{'}\mathbb{Y} - \mathbb{Y}^{'}\mathbb{X}\boldsymbol{\beta} - \boldsymbol{\beta}^{'}\mathbb{X}^{'}\mathbb{Y} + \boldsymbol{\beta}^{'}\mathbb{X}^{'}\mathbb{X}\boldsymbol{\beta} + \eta(\widehat{\dot{\mathbb{Y}}}^{'}\widehat{\dot{\mathbb{Y}}} - \widehat{\dot{\mathbb{Y}}}^{'}\mathbb{X}\boldsymbol{\beta} - \boldsymbol{\beta}^{'}\mathbb{X}^{'}\widehat{\dot{\mathbb{Y}}} + \boldsymbol{\beta}^{'}\mathbb{X}^{'}\mathbb{X}\boldsymbol{\beta}) + \lambda \sum_{j=1}^{p} \left\|\boldsymbol{\beta}_{(j)}\right\|_2 \\
=& \mathbb{Y}^{'}\mathbb{Y} + \eta\widehat{\dot{\mathbb{Y}}}^{'}\widehat{\dot{\mathbb{Y}}} - (\mathbb{Y}^{'} + \eta\widehat{\dot{\mathbb{Y}}}^{'})\mathbb{X}\boldsymbol{\beta} - \boldsymbol{\beta}^{'}\mathbb{X}^{'}(\mathbb{Y} + \eta\widehat{\dot{\mathbb{Y}}}) + (1+\eta)\boldsymbol{\beta}^{'}\mathbb{X}^{'}\mathbb{X}\boldsymbol{\beta} + \lambda \sum_{j=1}^{p} \left\|\boldsymbol{\beta}_{(j)}\right\|_2 \\
\propto& -(\mathbb{Y}^{'} + \eta\widehat{\dot{\mathbb{Y}}}^{'})\mathbb{X}\boldsymbol{\beta} - \boldsymbol{\beta}^{'}\mathbb{X}^{'}(\mathbb{Y} + \eta\widehat{\dot{\mathbb{Y}}}) + (1+\eta)\boldsymbol{\beta}^{'}\mathbb{X}^{'}\mathbb{X}\boldsymbol{\beta} + \lambda \sum_{j=1}^{p} \left\|\boldsymbol{\beta}_{(j)}\right\|_2 \\
\propto& -\frac{\mathbb{Y}^{'} + \eta\widehat{\dot{\mathbb{Y}}}^{'}}{1+\eta}\mathbb{X}\boldsymbol{\beta} - \boldsymbol{\beta}^{'}\mathbb{X}^{'}\frac{\mathbb{Y} + \eta\widehat{\dot{\mathbb{Y}}}}{1+\eta} + \boldsymbol{\beta}^{'}\mathbb{X}^{'}\mathbb{X}\boldsymbol{\beta} + \frac{\lambda}{1+\eta} \sum_{j=1}^{p} \left\|\boldsymbol{\beta}_{(j)}\right\|_2 \\
\propto& \frac{\mathbb{Y}^{'} + \eta\widehat{\dot{\mathbb{Y}}}^{'}}{1+\eta} \times \frac{\mathbb{Y} + \eta\widehat{\dot{\mathbb{Y}}}}{1+\eta} - \frac{\mathbb{Y}^{'} + \eta\widehat{\dot{\mathbb{Y}}}^{'}}{1+\eta}\mathbb{X}\boldsymbol{\beta} - \boldsymbol{\beta}^{'}\mathbb{X}^{'}\frac{\mathbb{Y} + \eta\widehat{\dot{\mathbb{Y}}}}{1+\eta} + \boldsymbol{\beta}^{'}\mathbb{X}^{'}\mathbb{X}\boldsymbol{\beta} + \frac{\lambda}{1+\eta} \sum_{j=1}^{p} \left\|\boldsymbol{\beta}_{(j)}\right\|_2 \\
=& \left(\widetilde{\mathbb{Y}} - \mathbb{X}\boldsymbol{\beta}\right)^{'}\left(\widetilde{\mathbb{Y}} - \mathbb{X}\boldsymbol{\beta}\right) + \frac{\lambda}{1+\eta} \sum_{j=1}^{p} \left\|\boldsymbol{\beta}_{(j)}\right\|_2,
\end{aligned}
\tag{S1}
$$

where $\widetilde{\mathbb{Y}} = (\mathbb{Y} + \eta\widehat{\dot{\mathbb{Y}}})/(1+\eta)$. Note that $L_{\lambda,\eta}(\boldsymbol{\beta};\mathbb{X},\mathbb{Y},\widehat{\dot{\mathbb{Y}}})$ is optimized to get an estimator of $\boldsymbol{\beta}$. Therefore, we can omit or add terms unrelated to $\boldsymbol{\beta}$ in the above deviation. For example, we omit $\mathbb{Y}^{'}\mathbb{Y} + \eta\widehat{\dot{\mathbb{Y}}}^{'}\widehat{\dot{\mathbb{Y}}}$ in the sixth line and add $\frac{\mathbb{Y}^{'}+\eta\widehat{\dot{\mathbb{Y}}}^{'}}{1+\eta} \times \frac{\mathbb{Y}+\eta\widehat{\dot{\mathbb{Y}}}}{1+\eta}$ in the eighth line.

S4 Computational algorithm for integrative analysis

We use the group coordinate descent technique to optimize the loss function. Denote $\mathbb{X}_{(j)}$ as the sub-matrix of $\mathbb{X}$ corresponding to the jth variable in the M datasets, i.e., $\mathbb{X}_{(j)} = \text{diag}(X_j^{(1)}, \ldots, X_j^{(M)}) \in \mathbb{R}^{n \times M}$. First, we orthogonize matrix $\mathbb{X}_{(j)}$ such that $n^{-1}\mathbb{X}'_{(j)}\mathbb{X}_{(j)} = I_M$. This can be done through singular value decomposition of the Gram matrix of the jth variable. Then the group coordinate descent technique is adopted. This technique applies an iterative strategy for coefficient updating. In each iteration, it optimizes the loss function with respect to one group of coefficients (i.e., $\boldsymbol{\beta}_{(j)}$). With fixed tuning parameters, we conduct the group coordinate descent-based optimization as follows. Denote the current estimate of $\boldsymbol{\beta}$ in the t-th iteration as $\boldsymbol{\beta}^{[t]}$. Then we can calculate the current residual as $\boldsymbol{r}^{[t]} = \widetilde{\mathbb{Y}} - \mathbb{X}\boldsymbol{\beta}^{[t]}$. For the j-th group of coefficients with $1 \leq j \leq p$, define $\boldsymbol{z}_{(j)}^{[t]} = n^{-1}\mathbb{X}'_{(j)}\boldsymbol{r}^{[t]} + \boldsymbol{\beta}_{(j)}^{[t]}$. We then update the estimate of $\boldsymbol{\beta}_{(j)}$ with the soft-thresholding operator $F\left(\boldsymbol{z}_{(j)}^{[t]}, \lambda/(1+\eta)\right)$, where function $F(\boldsymbol{u}, \lambda)$ is defined as:

$$F(\boldsymbol{u}, \lambda) = S(\|\boldsymbol{u}\|_2, \lambda)\frac{\boldsymbol{u}}{\|\boldsymbol{u}\|_2}, \tag{S2}$$

where $S(v, \lambda) = v - \lambda$ if $v > \lambda$, $S(v, \lambda) = v + \lambda$ if $v < -\lambda$, and $S(v, \lambda) = 0$ otherwise. Then we have $\boldsymbol{\beta}_{(j)}^{[t+1]} = F\left(\boldsymbol{z}_{(j)}^{[t]}, \lambda/(1+\eta)\right)$. Repeat the above steps for p covariates until convergence or reaching the maximum number of iterations. The whole algorithm is summarized below.

Algorithm S1 Group Coordinate Descent Algorithm for Integrative Analysis

Require: $\mathbb{X}$, $\widetilde{\mathbb{Y}}$, λ, η

Ensure: $\hat{\boldsymbol{\beta}}$

Initialize $t = 0$, $\boldsymbol{\beta}^{[0]} = (0, \ldots, 0)'$, and calculate the residual as $\boldsymbol{r}^{[0]} = \widetilde{\mathbb{Y}} - \mathbb{X}\boldsymbol{\beta}^{[0]}$;

repeat

    for $j = 1, \ldots, p$, update the jth coefficient group **do**

        Calculate $\boldsymbol{z}_{(j)}^{[t]} = n^{-1}\mathbb{X}_{(j)}'\boldsymbol{r}^{[t]} + \boldsymbol{\beta}_{(j)}^{[t]}$;

        Update $\boldsymbol{\beta}_{(j)}^{[t+1]} \leftarrow F\left(\boldsymbol{z}_{(j)}^{[t]}, \lambda/(1+\eta)\right)$;

        Update $\boldsymbol{r}^{[t]} \leftarrow \boldsymbol{r}^{[t]} - \mathbb{X}_{(j)}\left(\boldsymbol{\beta}_{(j)}^{[t+1]} - \boldsymbol{\beta}_{(j)}^{[t]}\right)$;

    end for

    Update $t \leftarrow t + 1$;

until convergence or reaching the maximum t

S5 Software development

To facilitate routine utilization by the broad community, we develop friendly software codes and make them publicly available at https://github.com/ldz7/PAIA. The screenshot of the github page is provided in Figure S3. Corresponding to the two steps of the proposed method, there are two software programs, with one for prior information extraction described in Section 2.2 (cnn_active_learning.py) and the other for prior information assisted integrative analysis described in Section 2.3 (PAIA.R). Researchers can use these two codes sequentially or separately.

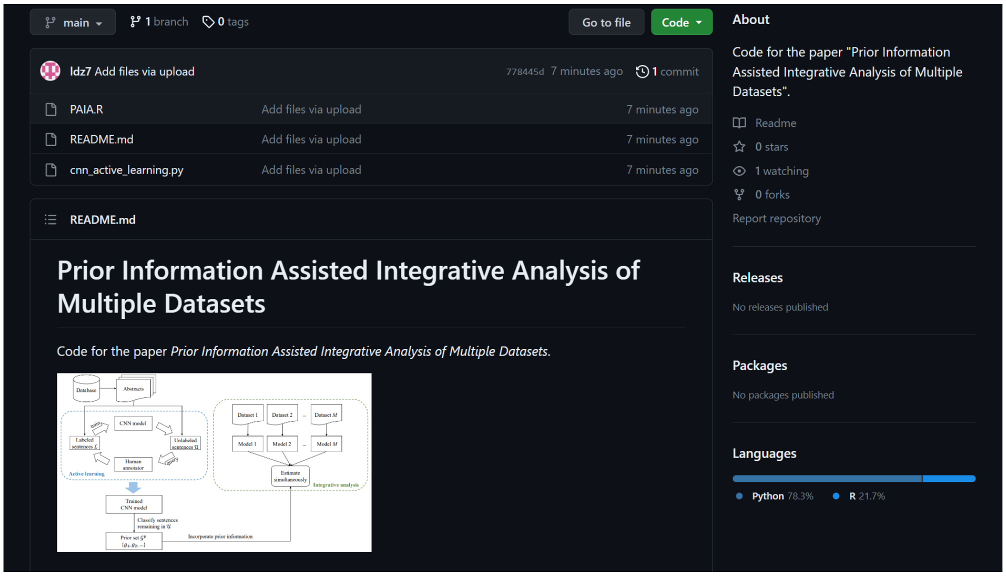

Figure S3: Software codes on GitHub.

S5.1 Prior information extraction (code *cnn_active_learning.py*)

Here, we implement the CNN model training with the active learning strategy. The code is written in Python 3.6, and some packages need to be installed before running (see the GitHub page for details).

Before running the code, researchers need to first download abstracts and split each

abstract into sentences, as described in the first two steps in Figure S2. As different ways are needed for abstract downloading from different databases, it is challenging if not impossible to provide a universal program. However, our own experience is that this can be relatively easily done.

After sentences are prepared, researchers need to reorganize them into an excel file with two columns ("sentence" and "relation") and put it in the same folder as the code. This file is the input of the code. The output of the code consists of multiple parts (see the GitHub page for more details), of which the most important two are the printed index (which indicates which sentences need to be labeled manually) and *al_metrics_dataset_name.xlsx* (which records model performance measured by AUC, sensitivity, specificity, and G-means in each activate learning iteration. *dataset_name* is the value of variable (*dataset* in the code). When the CNN model has reached a satisfactory result with active learning, we can load the trained model *model_iter=x.pt* (where x is the final active learning iteration) and use it to predict the labels of remaining sentences. Then the prior set $\mathcal{G}^p$ can be constructed by counting the number of sentences that are classified as related to the disease for each gene.

S5.2 Prior information assisted integrative analysis (code *PAIA.R*)

Here, we implement the proposed prior information assisted integrative analysis. The code is written in R 4.1.2, and some packages need to be installed before running (see the GitHub page for details).

The input of the code includes the prior set and datasets for integrative analysis. In each dataset, each row represents one observation, and each column represents one variable (covariates and response). The output is the estimated coefficients for different datasets. Here we note that although the code is designed for $M = 3$ datasets, it can be modified easily for other values of M.

S6 Additional Simulation Results

S6.1 Heatmaps of the estimated coefficients

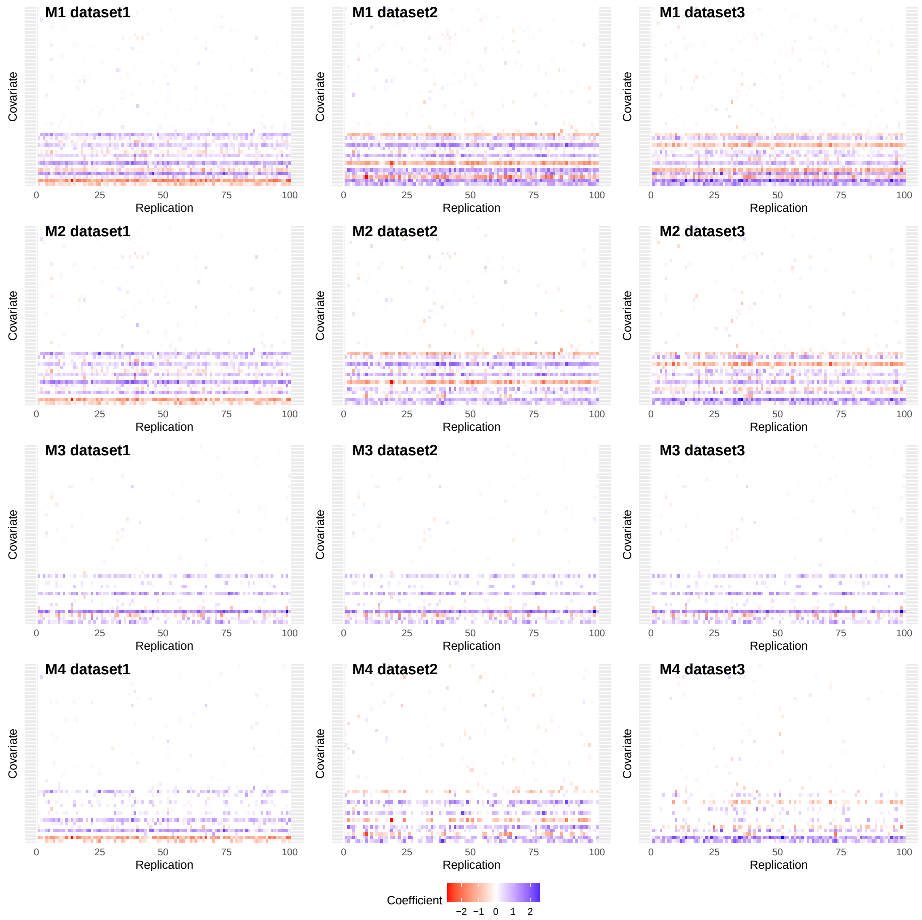

Figure S4: Coefficient estimation heatmaps under prior information set $\mathcal{G}_1^p$. In each heatmap, the rows correspond to covariates, and the columns correspond to data replicates.

S6.2 More settings of prior set

We consider two more settings of prior set: $\mathcal{G}_7^p = \{g_1, \ldots, g_{15}\} \cup \{g_{16}, \ldots, g_{95}\}$ and $\mathcal{G}_8^p = \{g_1, \ldots, g_{15}\} \cup \{g_{16}, \ldots, g_{210}\}$. Compared with $\mathcal{G}_4^p$, $\mathcal{G}_5^p$, and $\mathcal{G}_6^p$, the new settings $\mathcal{G}_7^p$ and $\mathcal{G}_8^p$ contain more relevant variables and also more irrelevant variables. As a result, the sensitivities of $\mathcal{G}_7^p$ and $\mathcal{G}_8^p$ increase, but the specificities decrease. We evaluate both variable

selection and prediction performance. The variable selection performance of $\mathcal{G}_7^p$ and $\mathcal{G}_8^p$ is summarized in Table S1. The corresponding results of $\mathcal{G}_4^p$, $\mathcal{G}_5^p$, and $\mathcal{G}_6^p$ can be found in Table 1. The prediction performance measured by MSE under $\mathcal{G}_4^p$ to $\mathcal{G}_8^p$ is reported in Table S2. Compared with $\mathcal{G}_4^p$ - $\mathcal{G}_6^p$, the new sets $\mathcal{G}_7^p$ and $\mathcal{G}_8^p$ have worse variable selection performance as measured by G-means and prediction performance as measured by MSE. This suggests that a prior set with lower specificity can worsen model performance. In particular, there is no difference between M1 and M2 under prior set $\mathcal{G}_8^p$. This result suggests that, when the prior set has too many irrelevant variables, prior information may not offer any assistance. Therefore, in prior information extraction, both sensitivity and specificity matter.

Table S1: Simulation results on variable selection.

Prior set	M1		M2		M3		M4	
	Mean	SD	Mean	SD	Mean	SD	Mean	SD
				Sensitivity				
$\mathcal{G}_7^p$	0.508	0.12	0.488	0.119	0.323	0.117	0.214	0.072
$\mathcal{G}_8^p$	0.488	0.119	0.488	0.119	0.215	0.092	0.208	0.074
				Specificity				
$\mathcal{G}_7^p$	0.994	0.005	0.994	0.005	0.993	0.006	0.993	0.004
$\mathcal{G}_8^p$	0.994	0.005	0.994	0.005	0.995	0.005	0.993	0.004
				Precision				
$\mathcal{G}_7^p$	0.619	0.153	0.621	0.158	0.506	0.209	0.379	0.139
$\mathcal{G}_8^p$	0.621	0.158	0.621	0.158	0.551	0.259	0.376	0.141
				G-means				
$\mathcal{G}_7^p$	0.706	0.084	0.691	0.084	0.556	0.104	0.455	0.078
$\mathcal{G}_8^p$	0.691	0.084	0.691	0.084	0.446	0.121	0.447	0.082

Table S2: Simulation results on prediction MSE.

Prior set	M1	M2	M3	M4
$\mathcal{G}_4^p$	18.263	21.859	27.218	24.347
$\mathcal{G}_5^p$	18.736	21.859	27.267	24.577
$\mathcal{G}_6^p$	20.976	21.859	27.766	25.957
$\mathcal{G}_7^p$	21.629	21.859	27.625	26.655
$\mathcal{G}_8^p$	21.859	21.859	28.100	26.751

S7 Discussion on publication bias

Publication bias has been well acknowledged and may have multiple aspects. For example, research with positive findings is more likely to be published. The attention that a finding (for example, an identified gene) received may not be “proportional” to its importance. Beyond bias, it is also recognized that published findings can sometimes even be wrong. All these factors can negatively affect the quality of prior information. Fortunately, they may not pose serious concerns. Specifically, in the proposed analysis, only information on identified genes is used. As such, the lack of published negative findings may not be a serious problem. In analysis, we only use information on whether there have been sufficient studies suggesting an association between a gene and the disease, as opposed to the count of published studies. This way, the disproportion problem mentioned above will not cause a serious problem. Additionally, a closer examination of the proposed approach suggests that it can screen out noises that have been falsely included in the prior information and identify signals that have not been included in the prior information. That is, it can flexibly accommodate both false positive and false negative information. To this end, we examine true and false positive (TP and FP) rates for covariates in and out of the prior information sets separately. The results are presented in Table S3. We find that, when some signals are missing from the prior set (e.g., $\mathcal{G}_1^p$ and $\mathcal{G}_2^p$), the proposed method can still identify them, and the TP rates are higher than the other three methods. When some noises are included in the prior set (e.g., $\mathcal{G}_4^p$, $\mathcal{G}_5^p$, and $\mathcal{G}_6^p$), only a small fraction of them will be identified. These results “re-confirm” the flexibility of the proposed approach.

Table S3: Simulation results for variable selection.

Prior set	M1		M2		M3		M4	
	Mean	SD	Mean	SD	Mean	SD	Mean	SD
TP rate (covariates in prior set)								
$\mathcal{G}_1^p$	0.887	0.099	0.556	0.168	0.438	0.206	0.517	0.114
$\mathcal{G}_2^p$	0.826	0.104	0.491	0.130	0.418	0.156	0.412	0.100
$\mathcal{G}_3^p$	0.827	0.097	0.488	0.119	0.416	0.130	0.354	0.089
$\mathcal{G}_4^p$	0.810	0.113	0.491	0.130	0.422	0.154	0.387	0.094
$\mathcal{G}_5^p$	0.784	0.129	0.491	0.130	0.421	0.154	0.377	0.089
$\mathcal{G}_6^p$	0.615	0.140	0.491	0.130	0.382	0.147	0.285	0.083
TP rate (covariates not in prior set)								
$\mathcal{G}_1^p$	0.527	0.109	0.453	0.125	0.150	0.088	0.204	0.069
$\mathcal{G}_2^p$	0.567	0.143	0.481	0.154	0.114	0.128	0.200	0.088
$\mathcal{G}_3^p$	0.000	0.000	0.000	0.000	0.000	0.000	0.000	0.000
$\mathcal{G}_4^p$	0.545	0.143	0.481	0.154	0.124	0.123	0.185	0.095
$\mathcal{G}_5^p$	0.525	0.145	0.481	0.154	0.126	0.123	0.186	0.092
$\mathcal{G}_6^p$	0.453	0.162	0.481	0.154	0.130	0.128	0.164	0.092
FP rate (covariates in prior set)								
$\mathcal{G}_1^p$	0.000	0.000	0.000	0.000	0.000	0.000	0.000	0.000
$\mathcal{G}_2^p$	0.000	0.000	0.000	0.000	0.000	0.000	0.000	0.000
$\mathcal{G}_3^p$	0.000	0.000	0.000	0.000	0.000	0.000	0.000	0.000
$\mathcal{G}_4^p$	0.087	0.104	0.013	0.038	0.060	0.108	0.058	0.067
$\mathcal{G}_5^p$	0.061	0.057	0.009	0.022	0.054	0.076	0.040	0.035
$\mathcal{G}_6^p$	0.019	0.019	0.008	0.012	0.050	0.043	0.018	0.013
FP rate (covariates not in prior set)								
$\mathcal{G}_1^p$	0.006	0.004	0.006	0.005	0.003	0.004	0.007	0.003
$\mathcal{G}_2^p$	0.006	0.002	0.006	0.005	0.002	0.003	0.008	0.004
$\mathcal{G}_3^p$	0.006	0.002	0.006	0.005	0.003	0.003	0.008	0.004
$\mathcal{G}_4^p$	0.006	0.003	0.006	0.005	0.003	0.003	0.007	0.004
$\mathcal{G}_5^p$	0.006	0.003	0.006	0.005	0.003	0.003	0.007	0.004
$\mathcal{G}_6^p$	0.006	0.005	0.006	0.005	0.004	0.004	0.007	0.004

S8 Additional data analysis results

S8.1 Implementation details of active learning

To apply active learning to label sentences, we first select 200 sentences and label them manually. These 200 labeled sentences are denoted by $\mathcal{L}_{training}$. Then we train a CNN classifier on $\mathcal{L}_{training}$. Next, in each step in active learning, we additionally label 100 sentences, which are identified as "difficult to be classified" by the classifier. The newly labeled sentences are added to $\mathcal{L}_{training}$, which is used to update the classifier. The prediction accuracy on a pre-labeled test dataset with 150 sentences is monitored. We repeat this step for 14 times. This results in a total of 200+100*14+150=1,750 sentences that are labeled manually. The prediction accuracy on the test data is 92.6%. It is noted that, some gene names (like KIT and APP) may have other meanings. Therefore, in the above labeling process, if we find gene names represent other meanings, we label the corresponding sentences as irrelevant.

S8.2 Boxplots of MSE

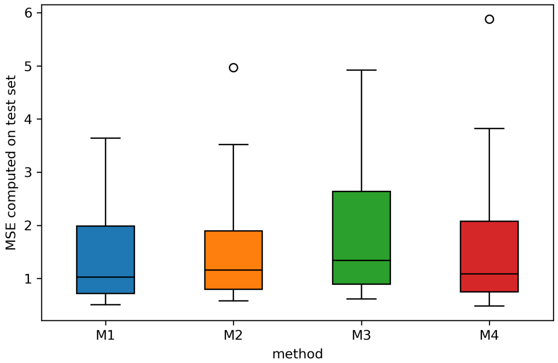

Figure S5: Data analysis: MSE values for 50 random splits.

S8.3 Gene selection results

Table S4: Data analysis: selected genes. Genes included in $\mathcal{G}^p$ are highlighted in bold.

Gene	M1 D1/2/3	M2 D1/2/3	M3 D1/2/3	M4 D1	M4 D2	M4 D3	Gene	M1 D1/2/3	M2 D1/2/3	M3 D1/2/3	M4 D1	M4 D2	M4 D3	Gene	M1 D1/2/3	M2 D1/2/3	M3 D1/2/3	M4 D1	M4 D2	M4 D3
AACS	✓				✓		HEXA					✓		PWWP2B			✓			
AARD			✓				HNRNPA0						✓	QRFP		✓	✓			
ABHD11.AS1			✓				HNRNPD			✓				RAB3D		✓	✓			
ADAM21P1						✓	HOXD11	✓		✓			✓	RAB9A					✓	
ADRB1	✓	✓				✓	IER5		✓					RBBP8NL		✓				✓
AGPAT3	✓						IGSF1			✓				RBFA					✓	
ALG2		✓				✓	IL13RA2			✓				RBPJ			✓			
ANAPC5		✓					IMPA2				✓			RCSD1	✓	✓	✓			
ANP32B					✓		IRAK1BP1						✓	RFX8			✓			
ANTXR2			✓				ITIH5		✓					RNF217						✓
ASB13						✓	JUN			✓			✓	RYBP	✓	✓				✓
B3GALT1		✓				✓	KCNA6						✓	SAMD15	✓					
**BAP1**			✓			✓	KCNK10					✓		SATB1			✓			
BEND5				✓			KCNMB2						✓	SDK2	✓	✓	✓			✓
BICD2				✓			KCNMB3		✓			✓		SEL1L3						✓
BLOC1S3			✓				KCTD16	✓	✓					SEMA3A	✓	✓	✓			✓
BOLA1	✓	✓	✓			✓	KCTD21	✓		✓				SERP2		✓	✓			✓
**BRAF**			✓		✓	✓	KCTD7		✓	✓		✓		SHISA3			✓			
BTD	✓	✓				✓	KDELR3	✓	✓				✓	SHOC2			✓			
BVES			✓			✓	**KIT**			✓		✓	✓	SLC12A9	✓	✓	✓			
C16ORF74	✓	✓	✓			✓	KLHL13	✓				✓		SLC24A2	✓		✓			
C1ORF216		✓	✓			✓	KPNA5		✓					SLC26A4					✓	
C8G			✓				LGALS3		✓			✓		SLC29A1					✓	
CARS						✓	LILRB1	✓						SLC35A2	✓	✓				
CCDC155					✓		LURAP1						✓	SLC8A1		✓				
**CD44**			✓				MACROD2		✓					SMARCD2	✓		✓			
**CDK4**	✓		✓		✓		MAFG						✓	SMC5						✓
CDKN1A	✓	✓					MAN1A1			✓				SMIM12	✓					
**CDKN2A**	✓	✓	✓			✓	MAP4K4	✓		✓		✓		SMNDC1		✓			✓	
CHP1	✓					✓	1-Mar		✓					SMPX		✓	✓			✓
CLDN11				✓			MBNL3		✓			✓		SNX22	✓	✓		✓		
CLDN16		✓				✓	**MC1R**	✓		✓		✓	✓	SOCS2			✓			
CLDND2			✓				MED30				✓			SOX8	✓	✓				✓
CNN3					✓		**MIA**	✓			✓	✓	✓	SPATA6					✓	
CNTN2		✓	✓			✓	MIPEP					✓		SPRYD7	✓	✓				✓
COL11A2	✓	✓				✓	MITD1				✓			ST6GAL1					✓	
COL9A2		✓					**MITF**	✓	✓	✓		✓	✓	STAM		✓	✓			✓
COLEC12		✓			✓		MPPE1						✓	STARD6	✓	✓				✓
CRACR2A		✓	✓			✓	MRPL47	✓	✓	✓			✓	SUMO2					✓	
CRTAP		✓	✓			✓	MSI1						✓	SYCE1L			✓			
CRYAA	✓	✓	✓			✓	MTHFSD	✓	✓				✓	TBC1D1		✓				✓
CSDE1			✓		✓		MUC5B	✓		✓				TCTA		✓				
CTNS	✓	✓			✓		MVB12B	✓						TDRG1	✓	✓				✓
**CXCR4**	✓	✓	✓		✓		NAALADL2		✓				✓	TEK			✓			
CXORF40A		✓					NAB2		✓			✓		TENT5C			✓			
CYTH3		✓					NASP					✓		**TERT**			✓		✓	
DAGLA	✓	✓	✓			✓	NATD1						✓	THAP2		✓				
DCHS1		✓					NAV1	✓					✓	TIAM2	✓	✓	✓			
DKKL1		✓					NDUFB5						✓	TIGAR	✓	✓			✓	
DMRT2	✓	✓			✓	✓	NFATC3		✓			✓		TLR4		✓				
DMRTA1						✓	NFIA					✓		TMBIM1	✓					
DNAL1	✓					✓	NHSL2			✓				TMEM100						✓
DOK1			✓				NIPA2						✓	TMEM120B						✓
DPYD	✓		✓				NME7	✓	✓	✓			✓	TMEM159	✓	✓	✓			✓
DTNB			✓				NPAS1					✓		TMEM185A	✓		✓			
DUSP12	✓	✓	✓				NPR1	✓	✓			✓	✓	TMEM233			✓			
DYNLRB2			✓				NR2F1	✓	✓	✓		✓		TMEM47			✓			
EBF1						✓	**NRAS**	✓		✓		✓	✓	TMEM74			✓			
EBF2	✓				✓		NXNL1			✓			✓	TMEM88			✓			
EBI3		✓	✓				NYAP2	✓	✓				✓	TMX2	✓					✓
EFCAB2			✓				OPN4	✓	✓				✓	**TNF**			✓			
EIF2B5	✓	✓	✓			✓	OR14C36	✓	✓	✓				TNFAIP1				✓	✓	
ELAVL4	✓		✓		✓	✓	OTOA	✓	✓	✓				TNFRSF4	✓	✓	✓		✓	
ELK3					✓		P4HA3		✓	✓				TOP1					✓	
ELOVL1	✓						PAQR7	✓	✓				✓	TPGS2	✓	✓				✓
EPB41L2						✓	PARD6G	✓	✓			✓		TRAF1			✓			
EYA4	✓	✓				✓	PEAR1		✓					TSHZ2					✓	
FAM107B		✓					PER1	✓		✓		✓		TSPAN16			✓			
FAM66D	✓		✓				PIP4K2C					✓		TTL		✓	✓			
FAM83B	✓						PITPNA		✓	✓		✓		TTYH2		✓				
FGF10	✓	✓	✓			✓	PIWIL2		✓	✓			✓	UCN	✓	✓	✓			
FMNL2						✓	PKD1L2	✓	✓	✓		✓	✓	ULK3	✓	✓				✓
FOXN4			✓				PLAGL1					✓		UPB1		✓				
GABRR2						✓	PLEKHO1					✓		UPK2	✓	✓	✓			✓
**GNA11**	✓		✓		✓	✓	PMS2P4	✓	✓	✓		✓	✓	UTRN		✓				
**GNAQ**	✓				✓		POMGNT1						✓	**VDR**			✓		✓	✓
GNAS						✓	POPDC2					✓		VOPP1		✓			✓	
GNB4			✓				**PRAME**	✓		✓			✓	VSTM4					✓	
GPR12			✓				PRMT6	✓	✓	✓		✓		ZCCHC3					✓	
GPR82						✓	PRMT8	✓	✓	✓			✓	ZDHHC9						✓
GREM2			✓			✓	PRSS22					✓		ZFHX3	✓					
GSDMD			✓				PRTFDC1		✓	✓				ZFYVE1		✓				
H3F3A			✓				PSRC1			✓				ZNF804B						✓
HCAR2		✓					**PTEN**			✓			✓	ZSCAN22			✓			✓
HELB					✓		PTTG2	✓	✓	✓			✓							
